# *N*-[4-Cyano-3-(trifluoro­meth­yl)phen­yl]-2-meth­oxy­benzamide

**DOI:** 10.1107/S1600536810050269

**Published:** 2010-12-18

**Authors:** S. Nanjunda Swamy, H. R. Manjunath, B. S. Priya, Basappa, M. A. Sridhar, K. S Rangappa

**Affiliations:** a Department of Biotechnology, Sri Jayachamarajendra College of Engineering, Mysore 570 006, India; b Department of Studies in Physics, Manasagangotri, University of Mysore, Mysore 570 006, India; c Department of Studies in Chemistry, Manasagangotri, University of Mysore, Mysore 570 006, India

## Abstract

In the title compound, C_16_H_11_F_3_N_2_O_2_, the carboxamide group connecting the two aromatic rings is in a *syn-periplanar* configuration; the mol­ecule is non-planar; the dihedral angle between the two aromatic rings is 13.95 (18)°. Intra­molecular N—H⋯O and C—H⋯O hydrogen bonds occur. In the crystal, mol­ecules are linked by inter­molecular C—H⋯O hydrogen bonds.

## Related literature

For nucleosome, a repeat unit of chromatin, see: Luger & Richmond (1998 ▶). For the biological activity of substituted amide derivatives, see: Bylov *et al.* (1999 ▶); Gududuru *et al.* (2004 ▶). For the preparation of the title compound, see: Mantelingu *et al.* (2007 ▶). For a related structure, see: Saeed *et al.* (2010 ▶).
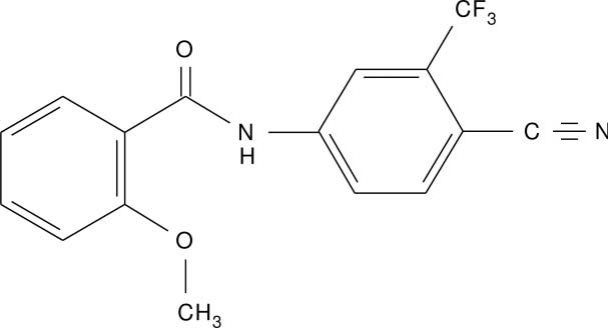


## Experimental

### 

#### Crystal data

C_16_H_11_F_3_N_2_O_2_
*M* *_r_* = 320.27
Monoclinic, 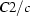

*a* = 15.117 (2) Å
*b* = 13.907 (2) Å
*c* = 14.5410 (11) Å
β = 107.360 (8)°
*V* = 2917.7 (6) Å^3^
*Z* = 8
Mo *K*α radiation
μ = 0.12 mm^−1^
*T* = 293 K
0.30 × 0.27 × 0.25 mm

#### Data collection

MacScience DIPLabo 32001 diffractometer
3420 measured reflections
1985 independent reflections
1623 reflections with *I* > 2σ(*I*)
*R*_int_ = 0.016
θ_max_ = 23.3°

#### Refinement

*R*[*F* ^2^ > 2σ(*F* ^2^)] = 0.060
*wR*(*F* ^2^) = 0.206
*S* = 1.03
1985 reflections
209 parameters
H-atom parameters constrained
Δρ_max_ = 0.30 e Å^−3^
Δρ_min_ = −0.21 e Å^−3^

### 

Data collection: *XPRESS* (MacScience, 2002 ▶); cell refinement: *SCALEPACK* (Otwinowski & Minor, 1997 ▶); data reduction: *DENZO* (Otwinowski & Minor, 1997 ▶) and *SCALEPACK*; program(s) used to solve structure: *SHELXS97* (Sheldrick, 2008 ▶); program(s) used to refine structure: *SHELXL97* (Sheldrick, 2008 ▶); molecular graphics: *PLATON* (Spek, 2009 ▶) and *ORTEPII* (Johnson, 1976) ▶; software used to prepare material for publication: *SHELXL97*.

## Supplementary Material

Crystal structure: contains datablocks global, I. DOI: 10.1107/S1600536810050269/jh2220sup1.cif

Structure factors: contains datablocks I. DOI: 10.1107/S1600536810050269/jh2220Isup2.hkl

Additional supplementary materials:  crystallographic information; 3D view; checkCIF report

## Figures and Tables

**Table 1:** Hydrogen-bond geometry (Å, °)

*D*—H⋯*A*	*D*—H	H⋯*A*	*D*⋯*A*	*D*—H⋯*A*
N7—H6⋯O15	0.96	1.90	2.648 (3)	133
C4—H12⋯O17^i^	0.96	2.41	3.346 (4)	165
C1—H15⋯O17	0.96	2.19	2.819 (4)	122

Symmetry code: (i) 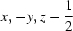.

**Fig. 1.**
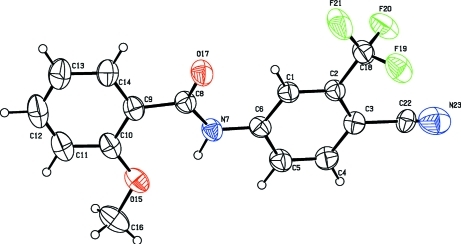
A view of the title compound, with 50% probability displacement ellipsoids.

**Fig. 2.**
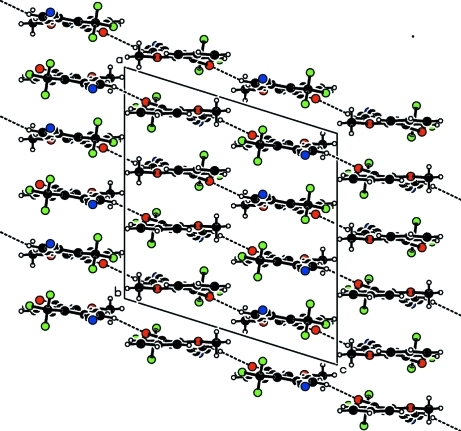
Packing diagram of the molecule viewed down the b axis. The dashed lines represents the hydrogen bonds.

## supplementary crystallographic information

### Comment

Nucleosome, a repeat unit of chromatin is made up of an octameric histone core, bearing two copies of H2A, H2B, H3 and H4 with 145–147 bp DNA wrapped around the central domain (Luger & Richmond, 1998). Several chromatin modifiers are reponsible for adding different post-translational marks like acetylation, methylation, phosphorylation and others on N-terminal histone tails and for dictating the degree of genomic compaction. Histone acetyltransferases add the acetyl group on the specific lysine of histone H3 and H4 N-terminal, and these signatures increase the accessibility of the underlying chromatin at specific genes or over vast regions of the genome. Compounds comprising an amide bond as backbone have a wide range of biological activities. Among the natural and synthetic substituted amide derivatives, there are compounds possessing anti-proliferative (Gududuru *et al.* 2004), anti-viral, antimalarial, general anesthetics, anti-inflammatory (Bylov  *et al.* 1999) and anti-microbial properties. In continuation of our research on benzamides, we have synthesized the title compound by the condenstation reaction and herein we report the single X-ray crystal structure of *N*-(4-cyano-3-(trifluoromethyl)phenyl)-2-methoxybemzamide.

A perspective view of the title compound is shown in Fig. 1. The carboxamide group connecting the two aromatic rings is in syn-periplanar-configuration. This is indicated by the torsion angle value of -7.6 (5)° about the atoms C6—N7—C8—O17. The two aromatic rings are out of plane with the dihedral angle value of 13.95 (18)° between the least squares planes of the rings. This value is very low when compare to the value of 57.69 (3)° (Saeed  *et al.* 2010) reported earlier. This can be understood in terms of the different substituents on the phenyl ring. The CN triple bond is affected by the *π*-delocalization which is evident from the value 0.854 (6)Å for C22—N23. The methoxy group attached to one of the aromatic ring lies within the plane of the ring and can be oriented in *trans* conformation. This is confirmed by the torsion angle value of 179.1 (3)° about the atoms C9—C10—O15—C16. The geometry around the C8 atom of the keto group is distorted trigonal as indicated by the bond angles of 120.6 (3)°, 122.4 (3)° and 117.0 (2)° for the atoms C9—C8—O17, N7—C8—O17 and C9—C8—N7, respectively. The crystal structure is stabilized by intermolecular C—H···O and intramolecular N—H···O, C—H···F and C—H···O hydrogen bonds. The intermolecular hydrogen bond C4—H12···O17 has the bond length of 3.346 (4)Å and the bond angle of 165° with the symmetry code *x,-y,-1/2 + z*. The molecules exhibit layered stackings when viewd down the *b* axis as shown in Fig. 2.

### Experimental

*N*-(4-Cyano-3-(trifluoromethyl)phenyl)-2-methoxybenzamide was synthesized as per the procedure reported in the literature (Mantelingu *et al.* 2007) earlier. The final product was obtained by recrystallization using methonol as a solvent. Slow evaporation of the solvent yielded colorless crystals after five days.

### Crystal data

**Table:**

C_16_H_11_F_3_N_2_O_2_	*Z* = 8
*M**_r_* = 320.27	*F*(000) = 1312
Monoclinic, *C*2/*c*	*D*_x_ = 1.458 Mg m^−^^3^
Hall symbol: -C 2yc	Mo *K*α radiation, λ = 0.71073 Å
*a* = 15.117 (2) Å	µ = 0.12 mm^−^^1^
*b* = 13.907 (2) Å	*T* = 293 K
*c* = 14.5410 (11) Å	Block, colorless
β = 107.360 (8)°	0.30 × 0.27 × 0.25 mm
*V* = 2917.7 (6) Å^3^	

### Data collection

**Table:**

MacScience DIPLabo 32001 diffractometer	1623 reflections with *I* > 2σ(*I*)
Radiation source: fine-focus sealed tube	*R*_int_ = 0.016
graphite	θ_max_ = 23.3°, θ_min_ = 2.3°
Detector resolution: 10.0 pixels mm^-1^	*h* = −16→16
ω scans	*k* = −15→14
3420 measured reflections	*l* = −14→14
1985 independent reflections	

### Refinement

**Table:**

Refinement on *F*^2^	Secondary atom site location: difference Fourier map
Least-squares matrix: full	Hydrogen site location: inferred from neighbouring sites
*R*[*F*^2^ > 2σ(*F*^2^)] = 0.060	H-atom parameters constrained
*wR*(*F*^2^) = 0.206	*w* = 1/[σ^2^(*F*_o_^2^) + (0.1344*P*)^2^ + 1.7982*P*] where *P* = (*F*_o_^2^ + 2*F*_c_^2^)/3
*S* = 1.03	(Δ/σ)_max_ = 0.003
1985 reflections	Δρ_max_ = 0.30 e Å^−^^3^
209 parameters	Δρ_min_ = −0.21 e Å^−^^3^
0 restraints	Extinction correction: *SHELXL97* (Sheldrick, 2008), Fc^*^=kFc[1+0.001xFc^2^λ^3^/sin(2θ)]^-1/4^
Primary atom site location: structure-invariant direct methods	Extinction coefficient: 0.006 (3)

### Special details

**Table:**

Geometry. Bond distances, angles *etc*. have been calculated using the rounded fractional coordinates. All su's are estimated from the variances of the (full) variance-covariance matrix. The cell e.s.d.'s are taken into account in the estimation of distances, angles and torsion angles
Refinement. Refinement on *F*^2^ for ALL reflections except those flagged by the user for potential systematic errors. Weighted *R*-factors *wR* and all goodnesses of fit *S* are based on *F*^2^, conventional *R*-factors *R* are based on *F*, with *F* set to zero for negative *F*^2^. The observed criterion of *F*^2^ > σ(*F*^2^) is used only for calculating -*R*-factor-obs *etc*. and is not relevant to the choice of reflections for refinement. *R*-factors based on *F*^2^ are statistically about twice as large as those based on *F*, and *R*-factors based on ALL data will be even larger.

### Fractional atomic coordinates and isotropic or equivalent isotropic displacement parameters (Å^2^)

**Table:**

	*x*	*y*	*z*	*U*_iso_*/*U*_eq_	
F19	0.08492 (17)	−0.25311 (16)	0.34995 (19)	0.1107 (10)	
F20	0.22717 (16)	−0.24074 (17)	0.37355 (18)	0.1129 (10)	
F21	0.1596 (3)	−0.14673 (16)	0.44676 (16)	0.1520 (15)	
O15	0.08614 (17)	0.31412 (17)	0.15351 (18)	0.0881 (10)	
O17	0.1218 (2)	0.15110 (16)	0.39923 (17)	0.0913 (10)	
N7	0.11310 (17)	0.14525 (17)	0.24161 (18)	0.0674 (9)	
N23	0.1364 (3)	−0.3157 (4)	0.1502 (3)	0.1214 (19)	
C1	0.1294 (2)	−0.0219 (2)	0.3009 (2)	0.0643 (10)	
C2	0.13454 (19)	−0.1182 (2)	0.2817 (2)	0.0619 (10)	
C3	0.1244 (2)	−0.1500 (2)	0.1890 (2)	0.0675 (11)	
C4	0.1073 (2)	−0.0828 (2)	0.1149 (2)	0.0765 (11)	
C5	0.1021 (2)	0.0126 (2)	0.1332 (2)	0.0734 (12)	
C6	0.11416 (19)	0.0455 (2)	0.2273 (2)	0.0626 (11)	
C8	0.1214 (2)	0.1927 (2)	0.3251 (2)	0.0666 (11)	
C9	0.1314 (2)	0.2998 (2)	0.3241 (2)	0.0698 (11)	
C10	0.1149 (2)	0.3588 (2)	0.2417 (3)	0.0763 (14)	
C11	0.1272 (3)	0.4564 (3)	0.2528 (4)	0.0975 (18)	
C12	0.1562 (3)	0.4967 (3)	0.3418 (4)	0.113 (2)	
C13	0.1740 (3)	0.4411 (3)	0.4235 (4)	0.1078 (19)	
C14	0.1608 (3)	0.3431 (2)	0.4140 (3)	0.0852 (14)	
C16	0.0674 (3)	0.3715 (3)	0.0683 (3)	0.1134 (19)	
C18	0.1520 (3)	−0.1878 (2)	0.3628 (2)	0.0781 (14)	
C22	0.1326 (3)	−0.2568 (2)	0.1652 (3)	0.0644 (12)	
H6	0.10490	0.18100	0.18320	0.0810*	
H9	0.09010	0.05830	0.08150	0.0880*	
H12	0.09920	−0.10360	0.05000	0.0910*	
H15	0.13630	−0.00070	0.36550	0.0770*	
H16	0.11520	0.49610	0.19650	0.1170*	
H19	0.17200	0.30390	0.47070	0.1020*	
H21	0.19460	0.46840	0.48700	0.1290*	
H22A	0.04740	0.33080	0.01260	0.1360*	
H22B	0.12280	0.40480	0.06770	0.1360*	
H22C	0.01970	0.41740	0.06730	0.1360*	
H23	0.16450	0.56510	0.34670	0.1360*	

### Atomic displacement parameters (Å^2^)

**Table:**

	*U*^11^	*U*^22^	*U*^33^	*U*^12^	*U*^13^	*U*^23^
F19	0.1193 (18)	0.0972 (16)	0.124 (2)	−0.0052 (13)	0.0490 (14)	0.0376 (13)
F20	0.1016 (17)	0.1068 (17)	0.1230 (19)	0.0267 (13)	0.0224 (13)	0.0395 (13)
F21	0.310 (4)	0.0802 (16)	0.0651 (16)	0.0290 (19)	0.0550 (19)	0.0146 (10)
O15	0.1043 (18)	0.0797 (16)	0.0830 (18)	0.0090 (13)	0.0323 (13)	0.0272 (12)
O17	0.150 (2)	0.0677 (15)	0.0623 (15)	−0.0031 (13)	0.0410 (13)	0.0051 (10)
N7	0.0877 (18)	0.0593 (15)	0.0600 (16)	0.0034 (12)	0.0296 (12)	0.0067 (11)
N23	0.123 (3)	0.149 (4)	0.097 (3)	−0.009 (3)	0.040 (2)	0.025 (3)
C1	0.0758 (19)	0.0645 (18)	0.0535 (17)	0.0015 (14)	0.0209 (13)	0.0019 (13)
C2	0.0686 (18)	0.0611 (17)	0.0582 (18)	0.0017 (13)	0.0222 (13)	0.0030 (13)
C3	0.0692 (18)	0.0673 (19)	0.069 (2)	−0.0013 (14)	0.0253 (14)	−0.0025 (14)
C4	0.102 (2)	0.079 (2)	0.0536 (19)	−0.0012 (17)	0.0312 (15)	−0.0052 (14)
C5	0.096 (2)	0.070 (2)	0.059 (2)	0.0031 (16)	0.0303 (15)	0.0081 (14)
C6	0.0683 (18)	0.0667 (19)	0.0567 (18)	0.0014 (13)	0.0245 (13)	0.0052 (13)
C8	0.0740 (19)	0.0647 (19)	0.065 (2)	0.0022 (14)	0.0268 (14)	0.0041 (14)
C9	0.0688 (18)	0.0625 (19)	0.085 (2)	0.0055 (14)	0.0334 (16)	0.0040 (15)
C10	0.069 (2)	0.0632 (19)	0.104 (3)	0.0077 (14)	0.0368 (18)	0.0155 (17)
C11	0.103 (3)	0.066 (2)	0.132 (4)	0.0093 (19)	0.048 (2)	0.020 (2)
C12	0.116 (3)	0.058 (2)	0.175 (5)	0.005 (2)	0.057 (3)	0.001 (3)
C13	0.120 (3)	0.075 (3)	0.133 (4)	0.000 (2)	0.045 (3)	−0.022 (2)
C14	0.096 (2)	0.068 (2)	0.096 (3)	0.0034 (17)	0.0353 (19)	−0.0104 (18)
C16	0.133 (4)	0.114 (3)	0.098 (3)	0.021 (3)	0.042 (2)	0.051 (2)
C18	0.099 (3)	0.0649 (19)	0.073 (2)	0.0073 (19)	0.0296 (17)	0.0068 (15)
C22	0.077 (2)	0.060 (2)	0.062 (2)	−0.0050 (18)	0.0294 (15)	−0.0145 (17)

### Geometric parameters (Å, °)

**Table:**

F19—C18	1.332 (5)	C8—C9	1.498 (4)
F20—C18	1.324 (5)	C9—C14	1.387 (5)
F21—C18	1.321 (4)	C9—C10	1.412 (5)
O15—C10	1.374 (5)	C10—C11	1.373 (5)
O15—C16	1.429 (5)	C11—C12	1.357 (8)
O17—C8	1.222 (4)	C12—C13	1.375 (7)
N7—C6	1.404 (4)	C13—C14	1.378 (5)
N7—C8	1.354 (4)	C1—H15	0.9600
N23—C22	0.854 (6)	C4—H12	0.9600
N7—H6	0.9600	C5—H9	0.9600
C1—C6	1.389 (4)	C11—H16	0.9600
C1—C2	1.375 (4)	C12—H23	0.9600
C2—C18	1.487 (4)	C13—H21	0.9600
C2—C3	1.383 (4)	C14—H19	0.9600
C3—C4	1.391 (4)	C16—H22A	0.9600
C3—C22	1.538 (4)	C16—H22B	0.9600
C4—C5	1.360 (4)	C16—H22C	0.9600
C5—C6	1.402 (4)		
			
F19···C22	2.982 (5)	C22···F20	2.948 (5)
F19···C3^i^	3.365 (4)	C22···F19^i^	3.230 (5)
F19···C22^i^	3.230 (5)	C22···F19	2.982 (5)
F20···C16^ii^	3.351 (5)	C8···H15	2.7500
F20···O15^ii^	3.060 (4)	C10···H6	2.6000
F20···C22	2.948 (5)	C11···H22C	2.7600
F20···F21^iii^	3.088 (4)	C11···H22B	2.7700
F21···F20^iii^	3.088 (4)	C13···H22C^i^	2.9900
F19···H22A^iv^	2.8100	C14···H22C^i^	3.0000
F19···H23^v^	2.8100	C16···H16	2.4900
F20···H23^v^	2.8500	C16···H6	3.0900
F21···H15	2.3200	H6···O15	1.9000
F21···H9^iv^	2.7700	H6···C10	2.6000
O15···N7	2.648 (3)	H6···C16	3.0900
O15···C9^i^	3.406 (4)	H6···H9	2.2300
O15···F20^vi^	3.060 (4)	H9···H6	2.2300
O17···C1	2.819 (4)	H9···F21^vii^	2.7700
O17···C4^iv^	3.346 (4)	H12···O17^vii^	2.4100
O15···H6	1.9000	H15···F21	2.3200
O17···H15	2.1900	H15···O17	2.1900
O17···H19	2.3900	H15···C8	2.7500
O17···H12^iv^	2.4100	H16···N23^viii^	2.7400
N7···O15	2.648 (3)	H16···C16	2.4900
N7···C8^i^	3.448 (4)	H16···H22B	2.2900
N23···H16^v^	2.7400	H16···H22C	2.2800
N23···H19^vii^	2.8200	H19···O17	2.3900
C1···O17	2.819 (4)	H19···N23^iv^	2.8200
C3···F19^i^	3.365 (4)	H21···H22B^ix^	2.5300
C4···O17^vii^	3.346 (4)	H22A···F19^vii^	2.8100
C4···C13^ii^	3.529 (6)	H22B···C11	2.7700
C5···C12^ii^	3.569 (6)	H22B···H16	2.2900
C8···N7^i^	3.448 (4)	H22B···H21^x^	2.5300
C9···O15^i^	3.406 (4)	H22C···C11	2.7600
C10···C10^i^	3.553 (5)	H22C···H16	2.2800
C12···C5^vi^	3.569 (6)	H22C···C13^i^	2.9900
C13···C4^vi^	3.529 (6)	H22C···C14^i^	3.0000
C14···C16^i^	3.557 (7)	H23···F19^viii^	2.8100
C16···C14^i^	3.557 (7)	H23···F20^viii^	2.8500
C16···F20^vi^	3.351 (5)		
			
C10—O15—C16	118.8 (3)	C9—C14—C13	121.3 (4)
C6—N7—C8	127.8 (2)	F19—C18—C2	112.6 (3)
C6—N7—H6	113.00	F20—C18—F21	107.2 (3)
C8—N7—H6	120.00	F20—C18—C2	113.5 (3)
C2—C1—C6	120.5 (3)	F21—C18—C2	113.4 (2)
C3—C2—C18	120.4 (3)	F19—C18—F20	103.2 (2)
C1—C2—C18	118.7 (2)	F19—C18—F21	106.1 (3)
C1—C2—C3	120.9 (3)	N23—C22—C3	178.3 (5)
C2—C3—C22	122.4 (3)	C2—C1—H15	120.00
C4—C3—C22	118.9 (3)	C6—C1—H15	119.00
C2—C3—C4	118.8 (3)	C3—C4—H12	120.00
C3—C4—C5	120.8 (3)	C5—C4—H12	119.00
C4—C5—C6	120.7 (3)	C4—C5—H9	120.00
N7—C6—C1	124.0 (3)	C6—C5—H9	119.00
C1—C6—C5	118.4 (3)	C10—C11—H16	119.00
N7—C6—C5	117.6 (2)	C12—C11—H16	120.00
O17—C8—C9	120.4 (3)	C11—C12—H23	119.00
O17—C8—N7	122.3 (3)	C13—C12—H23	120.00
N7—C8—C9	117.3 (2)	C12—C13—H21	122.00
C8—C9—C14	115.3 (3)	C14—C13—H21	119.00
C8—C9—C10	126.4 (3)	C9—C14—H19	119.00
C10—C9—C14	118.3 (3)	C13—C14—H19	119.00
C9—C10—C11	119.4 (4)	O15—C16—H22A	109.00
O15—C10—C11	123.4 (4)	O15—C16—H22B	109.00
O15—C10—C9	117.1 (2)	O15—C16—H22C	110.00
C10—C11—C12	120.9 (5)	H22A—C16—H22B	109.00
C11—C12—C13	121.1 (4)	H22A—C16—H22C	109.00
C12—C13—C14	119.0 (5)	H22B—C16—H22C	109.00
			
C16—O15—C10—C11	−0.3 (5)	C22—C3—C4—C5	−178.2 (3)
C16—O15—C10—C9	179.1 (3)	C2—C3—C4—C5	1.1 (5)
C8—N7—C6—C1	−3.7 (5)	C3—C4—C5—C6	0.2 (5)
C6—N7—C8—C9	171.7 (3)	C4—C5—C6—N7	176.5 (3)
C8—N7—C6—C5	178.5 (3)	C4—C5—C6—C1	−1.5 (5)
C6—N7—C8—O17	−7.6 (5)	N7—C8—C9—C10	13.5 (5)
C6—C1—C2—C18	179.3 (3)	O17—C8—C9—C10	−167.2 (3)
C6—C1—C2—C3	−0.2 (5)	O17—C8—C9—C14	13.6 (5)
C2—C1—C6—N7	−176.3 (3)	N7—C8—C9—C14	−165.8 (3)
C2—C1—C6—C5	1.5 (5)	C8—C9—C10—C11	180.0 (4)
C1—C2—C18—F21	0.8 (5)	C14—C9—C10—O15	179.8 (3)
C1—C2—C18—F19	121.3 (3)	C8—C9—C14—C13	179.2 (4)
C1—C2—C18—F20	−121.9 (3)	C10—C9—C14—C13	−0.2 (6)
C3—C2—C18—F21	−179.7 (4)	C14—C9—C10—C11	−0.8 (5)
C18—C2—C3—C4	179.4 (3)	C8—C9—C10—O15	0.5 (5)
C18—C2—C3—C22	−1.4 (5)	O15—C10—C11—C12	−179.7 (4)
C3—C2—C18—F20	57.6 (4)	C9—C10—C11—C12	0.9 (6)
C1—C2—C3—C22	178.1 (3)	C10—C11—C12—C13	−0.1 (7)
C3—C2—C18—F19	−59.2 (4)	C11—C12—C13—C14	−0.8 (7)
C1—C2—C3—C4	−1.1 (5)	C12—C13—C14—C9	1.0 (7)

Symmetry codes: (i) −*x*, *y*, −*z*+1/2; (ii) −*x*+1/2, *y*−1/2, −*z*+1/2; (iii) −*x*+1/2, −*y*−1/2, −*z*+1; (iv) *x*, −*y*, *z*+1/2; (v) *x*, *y*−1, *z*; (vi) −*x*+1/2, *y*+1/2, −*z*+1/2; (vii) *x*, −*y*, *z*−1/2; (viii) *x*, *y*+1, *z*; (ix) *x*, −*y*+1, *z*+1/2; (x) *x*, −*y*+1, *z*−1/2.

### Hydrogen-bond geometry (Å, °)

**Table:**

*D*—H···*A*	*D*—H	H···*A*	*D*···*A*	*D*—H···*A*
N7—H6···O15	0.96	1.90	2.648 (3)	133
C4—H12···O17^vii^	0.96	2.41	3.346 (4)	165
C1—H15···F21	0.96	2.32	2.673 (4)	101
C1—H15···O17	0.96	2.19	2.819 (4)	122
C14—H19···O17	0.96	2.39	2.729 (4)	100

Symmetry codes: (vii) *x*, −*y*, *z*−1/2.
